# Contributions of Genome Editing Technologies Towards Improved Nutrition, Environmental Sustainability and Poverty Reduction

**DOI:** 10.3389/fgeed.2022.863193

**Published:** 2022-03-17

**Authors:** Stuart J. Smyth

**Affiliations:** University of Saskatchewan, Saskatoon, Canada

**Keywords:** benefits, human health, innovation, sustainable development goals, yield increase

## Abstract

The Sustainable Development Goals (SDGs) were launched in 2015, with the top three goals being poverty eradication, improved food security and increased human health. All 17 SDGs have a target achievement date of 2030. These are ambitious and inspirational goals that require substantial innovation and technology adoption for successful achievement. Innovations in plant breeding have substantially contributed to transforming the efficiency of food production since the mid 20th century, with innovations emerging in the current millennium demonstrating enhanced potential to improve crop yields, the nutritional values of food crops and environmental impacts. These outcomes underpin several SDGs, but in particular the first three. As climate change is expected to become increasingly variable, with greater impacts on agriculture, the ability to ensure increased food production is going to be increasingly important, as higher yields directly contribute to reducing poverty. This article reviews recent reports of potential contributions from genome editing technologies in terms of increased yield, enhanced nutrition and greater sustainability, highlighting their importance for achieving the leading three SDGs.

## Introduction

Transformational innovations are rare occurrences within global economies. Some of the historical transformative innovations required several decades, or longer, to reach their full potential. The creation of the combustion engine, airplanes and computers are but a few examples of transformative innovations that had lengthy ramp up periods. The most recent transformative innovation, genome editing, is poised to have a relatively short ramp up period, with significant application being reported since its first discovery less than a decade ago (Doudna and Charpentier 2014). Genome editing is the ability to target and control mutagenic breeding technologies to a specific gene or genes, as compared to the insertion of genes utilized by genetic modification (GM) technologies. There are numerous genome editing technologies, such as clustered regularly interspaced short palindromic repeats (CRISPR), site directed nuclease and oligonucleotide-directed mutagenesis. These are genome site-directed technologies, capable of up-regulating, down-regulating or silencing a specific gene or genes.

Innovations and their systemic integration into farming practices have always been vital for agriculture and food production. Without the invention of synthetic fertilizers, farmers would face nutrient depleted soils. In the absence of herbicides, fungicides and insecticides, farmers would experience substantial yield losses from weeds, diseases and insects. Innovative plant breeding technologies like mutagenesis and GM have contributed to higher yielding varieties with strengthened pest resistance and stress tolerance traits. In combination, these innovations liberated farmers and consumers from ancient, food insecure crop and food production practices.

Innovations in other sectors of the agricultural industry have provided additional important technology advancements. The use of global positioning systems (GPS) has allowed farmers to minimize seed, fertilizer and spray overlaps, reducing input amounts required to produce a crop, resulting in greater farmer profitability. Advancements in seeding equipment have facilitated the transition away from the need to till the soil prior to seeding, as well as disturbing the soil while seeding. Modern drills and seeders are capable of sowing crops with virtually no soil disturbance, preserving soil moisture for seed germination. The scale of equipment has also significantly contributed to improved crop production, allowing farms to expand and increase economic efficiencies.

While innovations more than 100 years ago in chemicals and fertilizers began the transformation process in agriculture, it was not until the middle part of the 20th century that increases in production were decoupled from increases in land used for crop and food production (OECD 2021). Over the past 60 years, food production has increased 390%, while the amount of land used to produce food has only increased by 10%. This demonstrates the substantial impact the Green Revolution had beginning in the 1960s to identify innovative means of producing greater yields on the same amount of land. While all of the innovations outlined above have been important in this transition, perhaps none is more important than innovative technologies in plant breeding. Without investments into new and improved plant breeding technologies and their adoption, increased food production would still be reliant on increasing the amount of land used for food production. It is estimated that if GM crops had not been developed, commercialized and adopted, the 2018 production of canola, corn, cotton and soybeans would have required an additional 60 million acres of cropland, an area equivalent to 14% of cropland in the United States (Brookes and Barfoot 2020).

Breeding new plant varieties is not a simple, or rapid process, with development times of 12–15 years common. One estimate identifies that the application of genome editing technologies could shorten this time requirement to as few as 2–3 years (Friedrichs et al., 2019). One challenge of older plant breeding technologies was the accuracy of the resulting genetic changes. Mutagenic technologies introduce changes throughout the plant genome, affecting many genes, thus requiring substantial testing and breeding cycles to determine which changes proved advantageous. The advent of GM technologies allowed for improved breeding accuracy, with scientists able to introduce specific genetic traits with greater testability, such as herbicide tolerance or insect resistance. In the short time period of use, genome editing technologies offer further enhanced predictability and accuracy of modifying or deleting specific endogenous genes by making targeted genetic changes (or targeted mutations), as well as the ability to introduce new genes. This article examines the potential contributions with the wide-scale adoption of current applications of genome editing technologies in plant breeding towards achievement of the first three Sustainable Development Goals (SDGs).

In 2000, the United Nations (UN) established the Millennium Development Goals (MDGs)^1^ with a target achievement date of 2015. The first of the 10 MDGs was to eradicate extreme poverty, which is commonly defined as living on less than US$2/day. Significant progress towards this goal was achieved as nearly 200 million fewer people were identified as being undernourished in 2015, as compared to 2005 (FAO 2021). Households with higher levels of disposable income are able to allocate a greater percentage to food, contributing to lifting them out of extreme poverty and hunger. In 2015, the UN proposed 17 SDGs, with a target date of 2030.^2^ The first three SDGs expand on the first MDG and are identified as: 1) end poverty in all its forms, everywhere; 2) end hunger, achieve food security and improved nutrition and promote sustainable agriculture; and 3) ensure healthy lives and promote well-being for all at all ages.

### Genome Editing and the UN Sustainable Development Goals

With the application of many genome editing technologies in plant breeding being less than a decade old, few commercialized products have reached the market, making it a challenge to quantify the economic, environmental and nutritional benefits. Given that genome editing technologies are an evolution of existing mutagenesis and GM plant breeding technologies, it can be expected that they will further the benefits already observed and quantified with earlier technologies, when new crop varieties developed by genome editing become more commonplace (Ricroch et al., 2022). Furthermore, genome editing may overcome previous limitations of GM technologies, such as allowing improvement of existing local and regional crop varieties, rather than working on introducing new crop varieties into a region, with emerging evidence indicating that plant breeders are applying genome editing to local crops that both farmers and consumers are familiar with (Abdallah et al., 2015; Venezia and Creasey Krainer 2021). A significant benefit of genome editing technologies is that they can be seamlessly applied to existing variety development research programs, especially in developing countries.

Given the anticipated broader range of application of genome editing in breeding and the improved performance of genome editing compared to earlier breeding tools, it is relevant to examine genome editing in the context of innovations that contribute towards the achievement of the SDGs. The remainder of this section examines components of the first three SDGs that the application of genome editing in food crops has the potential to contribute to, concluding with the sub-section on poverty.

#### Genome Editing and Yield Increases

Multiple technologies, factors, policies and programs will be required to enable achievement of the set of SDGs, with agriculture being capable of anchoring much of the fundamental elements. Continuous improvement of crop breeding programs is crucial for increasing crop yields, leading to higher household incomes, which ultimately contributes to reduced poverty and SDG #1. This relationship has been quantified by research examining yield and profitability impacts of GM crops. In a review of the economic impacts resulting from the adoption of GM crops, 147 peer reviewed journal articles were analyzed, demonstrating that GM crop adoption resulted in yield increases of 22%, with farm profitability rising by a significant 68% (Klümper and Qaim 2014). A subsequent assessment of yield increases from GM corn adoption between 1996 and 2016 found increases of up to 24% (Pellegrino et al., 2018). An assessment of Bt brinjal in Bangladesh found yield increases of 20 and 22% higher revenues (Shelton et al., 2020). Over the past 20 years, evidence quantifying increased yields (along with safety and other benefits) resulting from the adoption of GM crops has continually accumulated, with estimates placing the total number of peer reviewed journal articles at over 3,000 (European Commission 2010; Nicolia et al., 2013; Van Eenennaam and Young 2014; National Academies of Sciences, Engineering, and Medicine 2016).

Breeding efforts to improve yields continue with the adoption of genome editing as a promising tool. To date, much of the global research investment into improved crop varieties has focused on the three global food staples: rice, wheat and corn. Rice plays an important role in the daily diets of people in many food insecure countries, often accounting as the sole source of nutrition. Considerable genome editing research has been directed at enhancing rice yield and improving other key traits. Researchers in China have been able to demonstrate increased yield and cold tolerance through the editing of just three genes (Zeng et al., 2020). Additional Chinese research has focused on improving disease resistance (Li et al., 2019). A global collaboration of researchers have focused on using genome editing to improve rice’s resistance to bacterial blight diseases, which reduces rice yields throughout Asia and Africa (Oliva et al., 2019).

Wheat is the second global food staple and genome editing is being applied to a wide range of traits. Considerable research is being undertaken focusing on increasing yields and improving both quality and disease resistance (Li et al., 2021). Other yield research is targeting increases in seed size and seed weight (Wang et al., 2018). With the sequencing of the wheat genome, genome editing is also being applied to enhance wheat traits based on those found in weedy relatives, thereby increasing the ability of new wheat varieties to better withstand changing climates (Pearce 2021).

The third staple for human food consumption to which genome editing is being applied is corn. The presence of mycotoxins in corn has long been an issue of high concern for its use in livestock feed and research is underway in the US utilizing genome editing to reduce mycotoxin levels (Bluhm and Swift 2019). Other American research focused on yield increases using genome editing has increased the number of rows of seed per cob (Cyranoski 2021). Genome editing is also being applied to develop semi-dwarf corn varieties that have equivalent yields but lower plant height, thereby lowering the amount of moisture and nutrients required to maintain yields (Bage et al., 2020). While the future of genome editing for plant breeding in Europe remains uncertain, researchers at Belgium’s Vlaams Instituut voor Biotechnologie (VIB) recently applied to conduct field trials involving three genome-edited corn varieties that have increased tolerance to climactic changes and improved digestibility (VIB 2022).

In a review of genome editing applications in plant breeding, Zhang et al. (2017) identify that research is globally proceeding in many other food crops besides the three staple crops discussed above, but also in citrus, vegetable and oilseed crops, with the leading traits including yield increases, increased disease and virus resistance and herbicide tolerance. Plant diseases can result in up to a 40% yield loss in food crops (Sastry and Zitter 2014) and genome editing technologies are providing advantages in mitigating these losses (Sharma et al., 2021). Wang et al. (2021) report that genome editing has been applied to improve disease resistance to fusarium wilt in bananas. Similar research is underway against fusarium wilt in Colombia (Maxmen 2019). Research applying genome editing technologies to cassava to improve resistance against cassava brown streak disease is also underway (Gomez et al., 2018).

With significant research underway utilizing genome editing to increase yields of the three global staple food crops, as well as other important food crops, genome editing technologies are demonstrating potential to substantially contribute to the components of SDG #2 focused on ending hunger and achieving food security. These applications are also important contributors to the component of SDG #2 focused on promoting sustainable agriculture (see the section on sustainability below).

#### Genome Editing and Nutritional Enhancements

Extensive research involving a variety of breeding technologies has been undertaken over the past 20 years on nutritional quality that includes: enhanced protein (canola, corn, potato, rice, wheat); increased oils and fatty acids (canola, corn, rice, soy); improved carbohydrates (corn, potato, sugar beet, soy); increased vitamins (potato, rice, strawberry, tomato); and increased mineral availability (lettuce, rice, soy, corn, wheat) (Newell-McGloughlin 2014). Early evidence is indicating that research on nutritional quality will benefit from genome editing technologies. It is also evident that public sector research institutions and laboratories are particularly active in this research. The application of GM technologies was predominantly confined to a small number of traits and multinational corporations due to the significant regulatory approval time and costs (Ku and Ha 2020).

Hefferon (2014) identifies that nutritional enhancement of the three staple crops has a lengthy period of research effort. Rice research has focused on addressing iron, folate and beta-carotene deficiencies (Majumder et al., 2019) as part of the effort to address micronutrient deficiencies, which are estimated to affect over 1.2 billion individuals globally and more commonly affecting children and women in developing countries (Wakeel et al., 2018). Numerous micronutrients, such as iron, zinc, selenium, magnesium, calcium and iodine, along with vitamins like provitamin A and folate play essential roles in the healthy development of children and the nutrition of nursing women and vegetables are increasingly the focus of research addressed at increasing these vital nutritional compounds (Lal et al., 2020). Israeli research involving genome editing applied to lettuce, has resulted in the creation of a new variety capable of expressing higher levels of beta-carotene, vitamin C and thiamine (Southy 2022). Vitamin A deficiencies annually results in over 250,000 instances of childhood blindness and contributes to other childhood and development problems like anemia, immune system deficiencies and stunted growth (Wesseler and Zilberman 2014; West and Darnton-Hill 2008). Ensuring that food nutrition is improved in childhood diets increases the capability of providing life-long benefits as individuals are less likely to have to manage debilitating diseases throughout their lifetime.

Another crop that is being utilized for increased micronutrient content is barley, which traditionally played a larger role in human food consumption, but has frequently been replaced by wheat in many modern diets (Sakellariou and Mylona 2020). Corn research has involved efforts to address beta-carotene, ascorbate and folate deficiencies. Biofortification research in wheat has improved zinc and selenium, both of which are important micronutrients. Research is additionally underway to enhance vitamin A content (Xiao et al., 2020) and provitamin A (Maqbool et al., 2018) in corn. In the United States (US), one focus of genome editing research in wheat has focused on fiber, which has led to field trials of a new high fiber wheat variety (Knisley 2021).

With genome editing technologies being broadly applied to nutritional enhancements in staple and other crops with promising results, particularly for food insecure developing countries, genome editing is poised to make significant contributions to the portion of SDG #2 that is focused on achieving improved nutrition. Improved nutrition additionally contributes to SDG #3, as the consumption of food with higher nutritional content contributes to healthier lives and improved well-being.

#### Genome Editing and Sustainability Improvements

While there are only a small number of genome-edited crops commercialized to date, they are demonstrating strong potential to both increase yields (see above) and improve agricultural sustainability (Qaim 2020). Field trial and experimental data show genome-edited crops can contribute to increased sustainability through improved water use efficiency (Glowacka et al., 2018) as well as nitrogen use efficiency (Wen et al., 2021). Research on improving yield, increasing fiber quality and enhanced environmental sustainability through enhanced drought tolerance of cotton is demonstrating how reductions in the environmental footprint of cotton production may be possible (Luo et al., 2019; Kumar et al., 2019; Peng et al., 2021). The environmental footprint of producing cotton can be reduced through integrating existing insect resistance and drought tolerance as these varieties will require fewer insecticide applications as well as lower amounts of water. Potato production requires extensive fungicide use and Norwegian research indicates that genome-edited potato varieties with increased resistance to late blight may be produced with significantly reduced volumes of fungicide applications (Turnbull 2021).

The application of genome editing technologies in agriculture has been dominated by plant research applications, but there have also been significant advancements in genome-edited meat. Leading the way is research in Japan that has resulted in the approval of two genome-edited fish for commercial sale (Nature Biotechnology 2021). Both the tiger puffer and the red sea bream fish have been edited to grow at a more rapid rate, on the same amount of feed. The tiger puffer is 90% heavier, while the red sea bream is 20% heavier. The ability to grow more rapidly reduces the amount of food required to raise each kilogram of meat, compared to non-edited fish. In a literature review of the application of genome editing technologies in aquaculture that focused on edits capable of contributing to improved sustainability, Blix et al. (2021) identify that China, Norway and the US have invested in this research. Changing climates are not restricted to land food production, with fish populations experiencing changes, contained aquaculture offers significant potential to ensure that fish supplies can continue to be a sustainable source of nutritional food in numerous countries.

Applications of genome editing in plants and livestock indicate that the technology is capable of contributing to the third component of SDG #2, promoting more environmentally sustainable agriculture. These applications also contribute to the other components of SDG #2 of ending hunger and achieving food security. Further advancements in increasing the sustainability of current food production practices are necessary in the face of pressures including climate changes and population growth and preventing conversion of more land from forest and other non-agricultural uses to agriculturally productive land. Without these areas of research focus, yields will decline with the effects of changing climates, which will adversely impact food security.

#### Genome Editing and Human Health

The third SDG focuses on ensuring that human health is promoted at all stages of life. It is well documented that GM crops have made substantial contributions to this through reductions in chemical exposure and improving food safety. For example, Kouser and Qaim (2011) estimate that as many as 9 million individuals per year in India no longer suffer from pesticide poisonings in the product of cotton following the adoption of GM varieties in 2003. Further, an assessment of over 20 years of GM corn production data identified that GM corn varieties exhibited 30% lower rates for the presence of cancer causing mycotoxins (Pellegrino et al., 2018). The use of genome editing may enable a more diverse scope of such benefits in an extended range of food crops.

An early example of this is a genome-edited tomato that has received commercial regulatory approvals in Japan. This product was developed to have increased levels of γ-aminobutyric acid (GABA) in the fruit, which may support lower blood pressure in consumers (Waltz 2021). In other tomato work in the US, genome editing is being used with a wild tomato variety from South America to increase lycopene, an antioxidant that has been linked to a lower risk of cancer and heart disease (Zaraska 2021).

In 2021, Rothamsted Research in the United Kingdom received approval for field trials of genome-edited wheat modified to produce less asparagine, a cancer causing compound when bread is toasted (Case 2021). Wheat consumption is problematic for people with gluten sensitivities or more serious celiac diseases. Genome editing has been applied to down regulate, silence and even delete gliadin genes, creating the potential for new wheat varieties that are safe for celiacs (Jouanin et al., 2020).

These applications of genome editing contribute to SDG #3, as they promote human health and well-being. When genome editing is capable of directly improving human health through the development of foods for consumption that may prevent specific diseases are integrated with applications that increase the nutritional values of foods, genome editing has the potential to make a substantial contribution to the achievement of SDG #3.

#### Genome Editing and Poverty

The combination of crops with higher yields and the resulting higher farm and household incomes contribute to poverty reduction goals. While a limited number of studies exist to date on this specific topic, one study has reported that the adoption of Bt cotton in India raised the income of adopting households living on less than US$2/day by 134% (Subramanian and Qaim 2010). This was achieved through a combination of higher yields and reduced input costs. The early evidence regarding the potential for yield increases using genome editing technologies indicates that, like GM crop adoption, their adoption could make significant contributions to the #1 SDG of ending poverty.

One proven means of reducing extreme poverty is through increasing the rate of education (Kulild 2014). Increases in household income provides opportunity for higher levels of child education. In some instances, children that are malnourished, are often unable to even attend school due to their physical weaknesses. Research on the importance of education and improved crop production, shows that an additional 4 years of education increases farm productivity by 8.7% (Lockheed et al., 1980). This is accomplished through a combination of higher education rates which increases farmers knowledge, awareness and importance of new methods of farming and food production and greater willingness to engage with, and accept the advice of, extension specialists.

### Regulation of Genome Editing

With the commercialization of genome-edited crops just beginning, regulatory frameworks range from non-existent to equivalent to GM crop regulations, which ultimately amounts to a ban on the technology. As of 2019, GM crops were produced in 29 countries and imported by a further 43, having undergone over 4,400 risk assessments in all countries since 1992 (ISAAA 2020). Many countries have not undertaken risk assessments of GM crops and still prohibit the import of these products.

Argentina and the US have been the global leaders on the regulation of genome editing technologies and products. In 2015, Argentina enacted new regulations for products of new breeding techniques, which included genome editing, becoming the first country to enact such regulations. Regulated on a case-by-case basis, the Argentine regulations stipulate that as long as no foreign DNA is present in the commercialized variety, the new variety will not be subject to additional regulatory oversight, thus being regulated as equivalent to conventional non-GM crop varieties (Whelan et al., 2020). In March 2018, the US Secretary for Agriculture issued a statement, indicating that the USDA would not regulate plants that were developed using any technology that resulted in a final variety that could have naturally developed and was not considered to be a potential plant pest (USDA 2018).

Argentina’s regulatory framework has been viewed by numerous other Latin American countries as reasonable and functional. Countries that have followed Argentina’s lead include: Chile, Brazil, Colombia, Paraguay, Ecuador, Honduras and Guatemala (Entine et al., 2021). Harmonized genome editing regulations will facilitate innovation and trade throughout Latin America, as barriers will be minimized due to variation in regulatory requirements between countries. Similarly, Canada has adopted genome editing regulations that align with its largest trading partner, the US. Both Health Canada and the Canadian Food Inspection Agency have indicated that genome-edited products are safe and if no foreign DNA is present in the final variety, these products will be exempt from regulatory oversight (Fawcett-Atkinson 2021). Presently, both regulatory bodies are revising existing regulations to enable these exemptions.

Australia has followed a similar path to Canada and the US, as it has stated that some genome editing technologies will not require additional regulation. Those technologies that are capable of inserting foreign DNA in the final product will require regulation as equivalent to a GM crop. As a result of this determination, some applications of site directed nucleases (SDN) are regulated as equivalent to GM crops (Entine et al., 2021).

Japan’s regulation of genome editing is similar to Australia’s, in that SDN technologies that do not contain inserted nucleic acid in the final variety will not be regulated under GM regulation, while those technologies capable of inserting foreign DNA will be regulated as utilizing GM regulations (Entine et al., 2021). Varieties containing inserted nucleic acid in the development process, but where it is removed in the commercialized variety, would not be regulated as equivalent to GM varieties.

Genome editing research exploded on the scene in China with the November 2018 announcement regarding the birth of twins that had been created from genome-edited embryos (BBC 2019). This announcement caught not only the world by surprise, but the Chinese government as well and resulted in a 3-year jail sentence for the lead scientist. As a result of this unsanctioned use of genome editing technologies, Chinese scientists involved in using these technologies as part of their plant breeding research were unsure as to what the government would decide and there was a period following the 2018 announcement with minimal application of genome editing. China has reaped significant benefits from GM cotton (Qiao and Huang 2020) and invests significantly in agriculture research, with one estimate of US$10 billion annually (Cohen 2019). As the regulations for genome editing became clearer, advancements in the use of genome editing technologies have been rapidly occurring as the Chinese government began to signal that genome editing technologies offered a viable solution for improving domestic food security (Patton 2022). Based on patent applications and those granted, there is substantial genome editing research underway in China (Turnbull et al., 2021). Given the significant investments the Chinese government has been making in agricultural research and with a clearer regulatory framework for genome editing technologies, it is reasonable to expect genome edited products to be entering the market within the next 5 years.

India was an early adopter of GM cotton, as 2022 marks its 20th year of production. However, India’s regulatory system has not approved any subsequent GM crop, as it has adopted Europe’s precautionary approach, resulting in gridlock that has prevented further GM crop commercialization. The lack of innovation in crop variety development has been very frustrating for Indian farmers as they observe farmers in neighboring countries increase profitability from the adoption of other GM crops. This has resulted in Indian farmers illegally growing GM brinjal, highlighting just how desperate Indian farmers are for innovative technology (Hindustan Times 2019). Given India’s political aversion to further commercial use of GM crops, adoption of genome editing technologies and the commercialization of any resulting products appears to be a long way from a reality.

Currently, the European Union (EU) is revising its regulations in relation to genome editing for plants with the objective to address the failures of its current GM framework which was identified as stifling innovation and not ‘fit for purpose’ by the EU Commission (European Commission 2021). While there is a concerted effort to effect regulatory reform for genome-edited crops, the changes that will be required to precipitate this will be contested and lengthy (Smyth and Wesseler 2022). It is important that the EU implement risk appropriate regulations for genome editing as the EU’s regulatory framework for GM crops was based on precaution, not risk, which had negative spillover effects on GM crop adoption in many developing countries (Paarlberg 2009; Smyth et al., 2016). Achievement of the SDGs by 2030 requires a more risk appropriate EU regulatory system.

Similar to how the EU’s GM regulatory framework affected regulation in African countries, the same is being observed with genome editing. South Africa has indicated that it will revise its regulatory framework to ensure that the application of genome editing technologies will result in them being regulated as equivalent to GM varieties (Government of South Africa 2021). The other two leading GM crop adopting countries, Kenya and Nigeria, are indicating they will not follow South Africa, rather the proposed regulatory revisions are inline with Argentina’s regulatory system (Entine et al., 2021). Nigeria is a regional influencer in terms of crop adoption and regulations and their position on genome editing regulation may encourage other nations in central Africa to adopt similar regulatory frameworks. For the most part, the regulation of genome editing in most African countries remains uncertain, as regulatory frameworks for GM technologies are lacking and by result, there are no regulatory frameworks for genome editing technologies either.

While the regulatory environment is evolving for genome editing, it is widely hoped that GM history will not be repeated and prevent realization of its potential, including application to local issues by agricultural research in developing countries. In many countries, GM regulations were not risk relevant and instead, were based on avoiding risk, which effectively resulted in bans on the application of GM technology. Developing countries will require innovative breeding tools if they are effectively able to respond to the SDGs. Application to local needs would include increased nutritional composition in local and regional varieties that farmers and consumers are familiar with. In addition to improved nutrition, applications such as improving disease resistance in local food crop varieties, improved drought tolerance and increased insect resistance would be of vital importance.

## Conclusion

The United Nations has charted an ambitious call for action by all countries through the launch of the SDGs, with an achievement target date of 2030. Agriculture has played, and will continue to play, a central role in achieving the SDGs, but the first three in particular: hunger eradication, improved food security and increased human health.

As reported by the OECD (2021), since 1960, food production has increased nearly four-fold, while the land used to produce this food has risen by only 10%. Driven by the Green Revolution, food production has vastly benefited from improved plant breeding investments and technologies. The widespread adoption of mutagenic plant breeding technologies to all crop variety development programs, which includes bulk commodities, fruits and vegetables, was a catalyst for the significant rise in food production. As the plant breeding technologies expanded to GM and now to genome editing, the ability to produce more per unit of land is expected to further increase, given that early evidence indicates higher rates of yield increases than previously achieved might be possible. The first of the commercial genome editing products and ongoing research in many areas of application also strongly suggest that a greater diversity of human health benefits may be possible. Regrettably, biotechnology-based agricultural innovations have long faced considerable opposition in parts of the world where the notion persists that older (pre-Green Revolution) food insecure production methods alone are the solution for improving food security, food quality and environmental impact. This is a dangerous fallacy and rejection of innovative plant breeding technologies, or insurmountable regulatory burdens imposed on them, will only perpetuate or worsen existing levels of food insecurity, malnourishment and adverse human health impacts, preventing progress towards the SDGs.

As production uncertainty increases in the face of changing climates, ensuring consistent yields and adaptable crops will be fundamental for improved food security and the societal benefits that arise from this. Modern plant breeding technologies have demonstrated significant successes in raising yields, increasing household wealth and improving food security. All plant breeding technologies will be required to further contribute to this and genome editing technologies may well be the 21st century drivers of food security, improved nutritional and human well-being and reduced poverty.

## Footnotes

1
https://www.un.org/millenniumgoals/.

2
https://sdgs.un.org/goals.
